# Suppression of TLR Signaling by Targeting TIR domain-Containing Proteins

**DOI:** 10.2174/138920312804871148

**Published:** 2012-12

**Authors:** Ota Fekonja, Monika Avbelj, Roman Jerala

**Affiliations:** 1 Laboratory of Biotechnology, National Institute of Chemistry, Ljubljana, Slovenia; 2 Centre of Excellence EN-FIST, Ljubljana, Slovenia and; 3 Faculty of Chemistry and Chemical Technology, University of Ljubljana, Ljubljana, Slovenia

**Keywords:** decoy peptides, inhibitory TIR domain-containing proteins, peptidomimetics, TLR signaling, therapeutic inhibition.

## Abstract

Toll-like receptors (TLRs) recognize molecules specific to pathogens and endogenous danger signals. Binding of agonists to the ectodomain of the receptor initiates TLR activation and is followed by the association of receptor cytosolic Toll/Interleukin-1 receptor (TIR) domains with TIR domains of adapter proteins leading to the assembly of signaling cascade of protein kinases that ultimately trigger the activation of transcription factors and expression of genes involved in the immune response. Excessive activation of TIR-domain mediated signaling has been implicated in inflammatory diseases (*e.g.* rheumatoid arthritis, systemic lupus erythematosus, colitis) as well as in the development of cancer. Targeting receptor-adapter interactions represents a potential strategy for the therapeutic TLR/IL-1R-specific inhibition due to the unique interacting domains involved. Peptide and protein-domain binding TLR inhibitors originating from the interacting surfaces of TIR-domain containing proteins can bind to the site on their target interacting protein thereby preventing the assembly of the functional signaling complex. Here we review protein-domain, peptide and peptidomimetic inhibitors targeting TIR-domain mediated interactions and their application demonstrated on *in vitro *and *in vivo* models. Recent structural data and elucidation of the molecular mechanisms of TIR-domain mediated signaling enabled the development of peptide inhibitors from TIR domains of TLRs and adapters, MyD88 intermediary domain as well as improved protein inhibitors based on TIR domain dimerization, mimicking bacterial TIR-domain containing immunosuppressors (TCPs) which we discuss with challenges concerning the delivery and specificity of inhibitors targeting TLR adapters.

## 1 INNATE IMMUNITY AND TLR SIGNALING

Innate immunity represents the first defense line of the host against pathogenic microbes providing the immediate immune response [1, 2]. Innate immune response is mediated by phagocytes such as macrophages and dendritic cells which discriminate between the self molecules and pathogens using pathogen recognition receptors (PRRs) which are able to detect the unique and conserved components of pathogens known as pathogen associated molecular patterns (PAMPs). Toll-like receptors (TLRs) are one of the largest and best-studied families of PRRs [3, 4]. Activation of TLR signaling pathways induces activation of the innate immune response by expression of defense molecules and primes the development of adaptive response [5, 6]. The 12 identified TLRs in mammals [7] exhibit a differential pattern of expression with respect to cell types as well as differential cellular localization. Cell surface TLRs recognize ligands derived from extracellular pathogens such as microbial membranous ligands peptidoglycan, lipopolysaccharide (LPS) and flagellin, whereas TLRs localized in intracellular compartments-TLR3, TLR7, TLR8 and TLR9-detect bacterial and viral nucleic acids released during intracellular infection [8]. 

TLRs share a conserved structure of ectodomain composed of a leucine-rich repeat domain (LRR), a transmembrane segment and a cytoplasmic Toll/IL-1 receptor domain (TIR). Upon interaction of specific PAMPs with the corresponding LRR of the receptor, TLRs undergo homo- or hetero-dimerization, which initiates their activation [9-12]. Downstream TLR signaling is mediated by the conserved cytosolic TIR domain [13-15] present not only in TLR/IL-1R but also in TLR adapter proteins which bridge the TLRs to the proteins of the subsequent signaling cascade leading to expression of genes involved in innate immune response. TLR signaling involves five adapter proteins: the myeloid differentiation factor 88 (MyD88), the TIR domain-containing adaptor protein inducing interferon-β (TRIF; known also as TICAM1), the MyD88-adaptor-like (MAL, known also as TIRAP), the TRIF-related adaptor molecule (TRAM; known also as TICAM2) and the sterile α- and armadillo-motif containing protein (SARM) [16]. Among them, MyD88 and TRIF function as signaling adapters that mediate interaction to downstream kinases, whereas TRAM [17, 18] and MAL/TIRAP [19, 20] act to translocate MyD88 or TRIF to the activated TLRs. On the other hand, adapter SARM functions as a negative regulator of TRIF signaling [21]. As shown on Fig. (**1**) two different intracellular pathways bifurcate from TLRs: adapter MyD88 dependent pathway that is employed by all TLRs except TLR3 [22-24] and TRIF dependent pathway [22, 25] used by TLR3 and TLR4. Except for TLR3, TLR activation recruits the adapter MyD88 into the receptor complex through TIR-TIR interactions followed by the recruitment of IRAK kinases mediated by DD-DD interactions and activation of TRAF6. Subsequent polyubiquitination of TRAF6 induces the activation of TAK1 which activates the IKK complex or MAP kinases leading to NF-κB or AP-1 induction and inflammatory cytokine transcription [4]. However, the engagement of TLR7 and TLR9 MyD88 dependent pathways induces not only the secretion of inflammatory cytokines due to the activation of NF-kB but also the expression of type I IFNs through the activation of IRF7 [26]. MyD88-independent pathway on the other hand engages adapter TRIF that activates NF-kB through the C-terminal part of TRIF interacting with RIP or through its N-terminal part through TRAF6 culminating in inflammatory cytokine production [27]. The activation of TRIF dependent pathway also results in the production of type I IFNs triggered by the activation of IRF3 induced by TBK1 kinase through the N-terminal part of TRIF [28, 29]. 

### 1.1 TLR Adapters and Their Structure

TLR signaling involves five adapter proteins containing TIR domains. MyD88 is the most ubiquitous adapter protein and is required for activation of signaling pathways specific to IL-1R and all TLRs except TLR3. MyD88 comprises death domain (DD) and TIR domain, which are linked by an intermediate segment or INT domain. TIR domain located at the C-terminus of MyD88 is required for binding to the activated receptor TIR domains, while the N-terminal DD is responsible for recruiting the IL-1R-associated kinase 4 (IRAK4) and for further propagation of the signaling cascade [30, 31]. An alternative transcript variant MyD88s (MyD88 short) that lacks the INT domain is transcribed in addition to wild type MyD88 [32]. MyD88s is unable to recruit the kinase IRAK4, although it contains DD and has been characterized as an inhibitor of MyD88 dependent signaling. MyD88s is induced by LPS and acts as an endogenous negative regulator of TLR signaling [33]. Mutations in the N-terminal part of INT domain diminish MyD88 signaling capacity [34]. The interactions of MyD88 with TIR domains of TLRs, IL-1R and DD of kinases are not yet explained at the molecular level. On the other hand the recent crystal structure of the complex of death domains of MyD88-IRAK4-IRAK2 describes the complex molecular assembly leading to the proximity between the kinase domains of IRAK4 and IRAK2/1 [35], however it cannot explain the role of TIR-TIR interactions. Based on our recent findings on the ability of the dimeric TIR domain for the initiation of signaling we proposed the molecular mechanism of TLR activation by a dimeric TIR platform. According to this model, the dimeric TIR domains of MyD88 represent the rate limiting step that initiates the assembly of the Myddosome [36]. The proposed molecular model with the potential inhibitory sites for peptide, peptidomimetics and protein inhibitors is represented on Fig. (**2**). 

The second discovered adapter was MAL/TIRAP. MAL/TIRAP comprises TIR domain at its C-terminus and a cationic phosphatidylinositol-4,5-bisphosphate (PtdIns(4,5) P2) binding segment located at the N-terminus. This cationic segment is required for its recruitment to the plasma membrane enriched in anionic lipids. It has been proposed that MAL/TIRAP assist in targeting MyD88 to the membrane TLR4 [20, 37, 38]. MAL-deficient mice demonstrated that MAL/TIRAP is essential for the sensitive MyD88 dependent signaling through receptors TLR2 and TLR4 [19] but not TLR5, although it is not essential for TLR2 activation. Crystal structure of MAL/TIRAP TIR domain identified important differences in the vicinity of the BB loop, important for the inhibition [39, 40]. 

Adapter TRIF contains a TIR domain, a receptor-interacting protein (RIP) homotypic interaction motif (RHIM) at its C-terminus and consensus TRAF6-binding motifs (T6BM) in the N-terminal region [41]. TRIF is required for TLR3 signaling and also mediates activation of TLR4. It has been demonstrated that TLR4 activation by TRIF dependent signaling pathway is delayed in comparison to the MyD88 dependent signaling, which has been proposed to be restricted to the plasma membrane while the internalized TLR4 activates TRIF dependent signaling from early endosomes [42].

Adapter TRAM, composed of a TIR domain at the C-terminus and an N-terminal myristoylation sequence, functions solely in the TLR4 pathway. N-terminus of TRAM undergoes constitutive myristoylation required for its interaction with membranes. It functions as a bridging adapter that recruits TRIF to TLR4 in TLR4 TRIF dependent signaling [43]. 

The adapter SARM also contains TIR domain at its C-terminus but acts as an inhibitor of TRIF dependent signaling. Besides TIR domain SARM contains two “sterile a” motif (SAM) protein-protein interactions domains and an Armadillo repeat motif (ARM) [16]. Recently, it has been proposed that SARM is related to bacterial TIR-containing proteins, suggesting that it differs in evolutionary origin from animal TIR-containing adapters. Similar to SARM bacterial TIR-containing proteins also negatively regulate eukaryotic immune response [44].

#### 1.1.1 TIR Domain

TIR domain is the central domain common to all five TLR adapters as well as TLR/IL-1R receptors. The global fold of the determined structures of TIR domains of TLR1, TLR2 [45], TLR10 [46], IL-1RAPL [47] and MyD88 [30] reveals the structure of five-stranded parallel β sheets (βA-βE) and five helices (αA-αE) connected by surface-exposed loops. TIR domain contains three highly conserved sequence motifs designated Box 1, 2 and 3. Sequence conservation among the TIR domains is generally only between 20 and 30% and their size ranges between 135 and 160 residues [45]. This sequence and structural diversity determines the specificities of receptor and adapter TIR-TIR interactions which defines the formation of initial signaling complexes in the TLR pathway [38, 45]. Multiple interaction surfaces of the TIR domain have been proposed to mediate the TLR oligomerization, association between the receptor and adapter TIR domains and adapter oligomerization [45]. TIR domains of TLR4 exhibit the constitutive interaction, which is inhibited by ectodomain, making it responsive to the agonist [48]. 

Several studies pointed out the importance of the conserved BB loop that connects the βB sheet to αB helix for TLR signal transduction [45]. BB loop contains an invariant proline residue that was shown to be essential for the signaling function of TLR2 [49], TLR4 [50], MAL/TIRAP [20] and TRAM [51]. Mutation of this proline to histidine in TLR4 (murine P712H) leads to hyporesponsiveness of C3H/HeJ mice to lipopolysaccharide [50] and the mutation of the corresponding proline within the TLR2 disrupts signaling induced by Gram-positive bacteria [49]. The lack of interaction of these two mutants with adapter MyD88 [45, 52] was proposed as the cause for their functional impairment. Mutations in the vicinity of this invariant proline residue inside the BB loop also diminished signaling of TLR4 and TLR2 [45, 53]. Similarly, mutations in the BB loop of MyD88 close to conserved proline (P200) are important for the interaction with IL-1 RAcP [54] however the P200H mutation of MyD88 retains the ability to activate NF-κB [20] and interaction with TLR4 [38] and IL-1 RAcP [54]. The decreased activity of the conserved proline mutation of TLR10 correlates with the position of this mutation on the TIR interaction surface as observed in the crystal structure of TLR10 TIR domain dimer which represents the first structural evidence for the existence of a TIR domain dimer with a putative signaling function [46]. Different molecular models for heteromeric TIR-TIR interactions have been proposed based on the data from crystal structures and mutational analyses which also support the importance of the BB loops in TIR domain interactions [54-56]. 

## 2 INHIBITION OF THE TLR SIGNALING

### 2.1 Implication of the TLRs in the Diseases

TLR activation is required for the defense against microbial infections but on the other hand TLRs are involved in the development of many other noninfectious diseases. Dysregulated or excessive response to bacterial endotoxin recognized by the TLR4/MD-2 (myeloid differentiation factor 2) complex results in the systemic inflammatory reaction which often leads to sepsis with high mortality [57]. Besides PAMPs, TLRs are also able to recognize endogenous molecules released by the damaged tissue, whose identity however has not been fully established. TLR recognition of the endogenous ligands results in sterile inflammation, which can lead to the development of chronic inflammatory and autoimmune diseases. Sterile inflammation is characterized by the accumulation of immune cells and release of lipid mediators, cytokines and harmful enzymes that can cause additional tissue damage [58, 59]. The involvement of the TLR signaling in rheumatoid arthritis (RA) was shown on mice lacking MyD88 or TLR2 that were resistant to the induction of the disease [60]. Mice lacking TLR2 or TLR4 produce less inflammatory cytokines in ischemia/reperfusion (I/R) and develop less severe inflammation [61, 62]. Mice deficient in MyD88 are less prone to atherosclerosis [63, 64] and patients with D299G polymorphism of TLR4 have reduced risk of this disease [65]. TLR7 and TLR9 receptors of pDCs are activated by immune complexes of autoantibodies coupled to host DNA/RNA and contribute to the development of systemic lupus erithematosus (SLE) [66]. Autoreactive B cells in SLE can also be activated directly through TLR7 and TLR9 [67]. Uric acid crystals activate NLRP3 inflammasome leading to production of IL-1β, which subsequently activates IL-1R/MyD88 pathway shown to be critical for elastase-induced lung inflammation and emphysema [68] as well as for gouty inflammation [69, 70]. MyD88 KO mice are protected from the development of type I diabetes [71] and neuronal MyD88 dependent signaling is involved in the diet-induced leptin and insulin resistance and obesity [72]. TLR4 plays a pivotal role in alcohol-induced neuroinflammation and brain damage [73] and the involvement of TLR/IL-1 signaling has been demonstrated for several other neurodegenerative diseases including multiple sclerosis [74], Alzheimer’s disease [75] and in the development but also in therapy of cancer [76-78]. 

### 2.2 Negative Regulation of TLR Signaling

In order to maintain the balance between host-defense functions and harmful effects leading to the aforementioned diseases, activation of TLRs needs to be tightly regulated. Therapeutically, TLR activation may be inhibited with TLR antagonists, neutralising antibodies to TLR ectodomains, small molecules that block enzymes in the signaling pathway such as IRAK4 and finally agents that block protein-protein interactions in the TLR signaling cascade. Great deal of effort has been invested particularly into the development of TLR4 antagonists for sepsis [79, 80] and antibodies against TLR2 or TLR4 have shown some promising results for the therapy of sepsis [81]. TLR9 and/or TLR7 antagonists [82, 83] and suppressive ODNs [84] may provide therapeutic benefits to SLE. Much attention has been dedicated to the inhibition of intracellular signaling targeting NF-κB, including inhibitors of the IκB degradation, inhibitors of the IKKβ [85] as well as inhibitors directed against MAP kinases [86]. Structural specificities of the kinase domain of IRAK4 opens the possibilities for specific inhibitors of this kinase [87]. Since the IKK and MAPK cascades are not only involved in the inflammatory processes but also have other important biological functions, side effects of these therapeutics have to be considered.

Recent findings on the role of adapter proteins and their interactions in TLR signaling open new possibilities for the development of selective inhibitors at the level of receptor-adapter interactions. Interactions between proteins involving TLR adapters also represent targets for endogenous host TLR regulation and for pathogen-mediated suppression of TLR activation, such as already mentioned MyD88s [33] and SARM [21]. The TIR-domain containing orphan receptors ST2 and SIGGIR inhibit TLR4 and IL-1R signaling [88, 89], while the SOCS1 regulator triggers degradation of the adapter MAL/TIRAP [90]. 

Pathogens have acquired the ability to interfere with the adapter-receptor TIR:TIR interactions of the host TLR/IL-1R signaling by means of TIR-domain containing proteins (TCPs) [91-93]. Protein NS3/4A of the hepatitis C virus cleaves the adapter TRIF in order to prevent the interferon type I production [54]. 

In contrast to the inhibition of TLR4 signaling, where the inhibition can be directed against the lipid A binding pocket of MD-2 [80, 94-97], screening of chemical libraries comprising small molecules for TLR inhibition has not resulted in compounds that are able to block protein-protein interactions due to the flatness of the interaction surfaces and absence of deep cavities appropriate for small molecule drugs binding [59]. However, targeting TLR and adapter interactions represents a promising potential for the specific TLR inhibition due to the interacting domains involved that are unique to the TLR signaling pathway, such as the TIR domain. Structural studies of these domains enabled the development of peptides and peptidomimetics from the interaction surfaces that occupy the docking site of the cognate protein on its target and prevent binding of the native protein thereby disrupting formation of the functional signaling platform. Similarly, protein inhibitors based on the TIR interacting domain interweave and compete with TIR:TIR interactions in the signaling complexes and thereby represent a promising therapeutic potential. This review limits its scope to the peptides, peptidomimetics and proteins that originate from the interacting domains of TIR-domain containing proteins and target their interactions. 

### 2.3 Inhibitors Originating from TIR-Domain Containing Proteins

#### 2.3.1 Inhibitory Peptides and Peptidomimetics

Since the infection or sterile inflammation is typically accompanied by activation of more than one TLR, it is important to inhibit signaling of several TLRs, which can be accomplished by targeting TIR domain or downstream signaling interactions rather than TLR ectodomains. This section focuses on peptides and peptidomimetics/small molecules targeting interactions among the adapters or between the adapters and receptors. To efficiently block intracellular targets, therapeutic agents must first enter into the cell, therefore polar peptides require additional cell-penetrating moieties to cross the membrane. 

##### 2.3.1.1 Cell-Penetrating Moieties

Two types of internalization motives have been added to inhibitory peptides targeting TLR signaling - the cell-penetrating peptides (CPPs) or protein transduction domains (PTDs), which are short cationic peptides, enriched in basic amino acids such as arginine or lysine, while the second approach is coupling fatty acids to peptides. 

Several naturally occurring CPPs are known, such as TAT peptide from HIV-1 virus and a sequence of homeodomain of the transcription factor antennapedia in Drosophilidae Antp43-58 also known as penetratin, while many such peptides have been artificially designed, *e.g*. hexa-arginine [98]. Internalization efficiency of CPPs depends on both the peptide length and its sequence. Six to eight residue polyarginine peptide is sufficient for translocation into mammalian cells [99]. The presence of specific sequences also enables CPPs to translocate into the specific organelles, *e.g*. nucleus [100].

CPPs conjugated with biologically active peptides or proteins have the ability to internalize into different cell types. Cells internalize these complexes in a concentration dependent manner and the highest intracellular concentration can be usually reached within 15 min. The biological activity of internalized proteins was detected even at low nanomolar concentrations. For complexes with TAT peptide or penetratin effective *in vivo* internalization in mice has been shown [101, 102]. The mechanism of internalization of cationic CPPs into the cells is still unclear. In contrast to many membrane-associating peptides that translocate across membranes by pore formation and are therefore toxic, toxicity was not observed for CPPs such as penetratin, TAT or polyarginine sequences making them safer for *in vivo* use [98, 103, 104]. The generally accepted mechanism of CPP internalization involves endocytosis, although it has been reported that CPPs can directly translocate through the membrane when the endocytosis is inhibited. TAT peptide is able to form interactions with several cellular components that allow it to translocate through the membrane with or without receptors [99, 104]. 

Less known and for the purposes of inhibition of cell signaling only recently used approach is to transport peptides into the cell using conjugation of a fatty acid chain [34, 105]. Myristoylation was efficient for the peptide uptake even in cell lines like the B lymphocyte cell line BA/F3 which is resistant to the peptide uptake using CPP derived from TAT peptide [105]. It has been shown that myristoylated cargo reaches its maximal intracellular concentration and biological activity within 30 minutes [34, 105]. In contrast to TAT peptide that internalizes efficiently at 37°C or 4°C, experiments performed with myristoylated cargo demonstrated temperature dependence in the cellular uptake which is thus more efficiently translocated at 37°C [105]. The myristoylated peptides also do not affect cell viability at concentrations up to 100 μM demonstrating its suitability for *in vivo* studies. Since the penetratin was shown to have an inhibitory effect on NF-κB signaling at higher concentrations [106], addition of fatty acid could become a more common cell-translocation motif.

##### 2.3.1.2 Inhibitory Peptides Targeting TLR Adapter/ Receptor Interactions

The adapters of TLR signaling represent more narrow targets for the inhibition of the inflammatory response in comparison to the receptors. In particular, the BB loop region within TIR domain and INT domain of MyD88 represent a segment of the functional interface of TIR domain critical for the proper signaling and a segment important for the interaction with IRAK4 and downstream propagation of signaling, respectively [see Fig. (**3**)] [34, 45, 50]. Several “decoy peptides”, corresponding to the surface exposed regions of TIR domains probably participating in interactions with other TIR domains have been investigated. Besides the internalization moiety decoy peptides comprise the polypeptide segment of the binding partner of the target protein [Table (**1**)] and mimic its interaction surface to prevent interaction of target protein with its counterpart. 

Research group from the University of Maryland School of Medicine developed several decoy peptides from TIR domain of adapters or receptors and found that inhibitory peptides comprising penetratin as an internalization motif and a sequence corresponding to the BB loop of adapters MyD88, MAL/TIRAP, TRIF and TRAM inhibit TLR4 signaling [107-109]. Although BB loop peptides can bind to proteins containing TIR domains, they do that with variable affinity and as such lack the desired specificity for targeting *e.g*. only MyD88 dependent TLRs or a specific TLR. The same research group also designed a set of decoy peptides with added penetratin which entirely tile the TLR4 TIR domain surface [110]. Five of these peptides derived from area between BB loop of TLR4 and its fifth helical region inhibited TLR4, but not TLR2 signaling suggesting this area mediates TLR4 TIR:TIR dimerization. More recently in a similar study peptides derived from the MAL/TIRAP have been tested for the ability to inhibit TLR2 and TLR4 signaling. Five MAL/TIRAP decoy peptides inhibited LPS-induced TLR4 signaling and two inhibited signaling induced by a TLR2/TLR1 agonist. Comparison of TLR4 inhibition by peptides derived from the TIR TLR4 or MAL/TIRAP indicate that structurally diverse regions mediate functional interactions of TIR domains [111]. Besides experiments on cellular cultures, some of these peptides were administrated in *in vivo* studies, for example a BB loop peptide from MAL/TIRAP attenuated LPS-induced lung response in C57BL/6 mice [112]. 

BB loop of TIR domain was a focus of interest also for group from the University of Rome “Tor Vergata” [113]. By *in vitro* assays Loiarro *et al.* showed that peptides derived from BB loop of MyD88 and IL-18R TIR domain effectively inhibit *in vitro* homodimerization of MyD88 TIR domain. Besides that, in cell activation assays this peptide fused to the penetratin peptide significantly reduced the IL-1R signaling [114]. MyD88 homodimerization inhibitory peptide also led to decreased growth in four different murine mammary carcinomas as well as in the human breast cancer cell line providing evidence that MyD88 is important for growth and metastasis of breast cancer [115].

Besides TIR derived inhibitory peptides, peptide derived from the N-terminus of the INT domain of MyD88 (called INT peptide) inhibited signaling of several MyD88 dependent TLR receptors as well as IL-1R. Its specificity was determined by the lack of inhibition of MyD88 independent signaling of TLR3 and TNF-R. In addition to the penetratin as internalization moiety, INT peptide was the first signaling inhibitory peptide to use myristoylation as an alternative cell penetration moiety. INT peptide was shown to bind to IRAK4 suggesting that INT domain of MyD88 is important for MyD88 signaling and interaction with IRAK4. Furthermore, INT peptide inhibited production of inflammatory cytokines *in vivo* and improved survival on the endotoxaemic mouse model [34]. 

Another somewhat different example of inhibitory peptides is a VIPER peptide derived from vaccinia virus protein A46 with polyarginine added as internalization moiety. Several viral proteins target host proteins using evolutionary optimized binding surfaces [116]. It has been shown that protein A46 binds to several TIR-domain containing proteins and as such prevents TLR-induced NF-kB, MAPK, and IRF3 activation [117]. VIPER, 11 aminoacid long peptide from A46, inhibited specifically TLR4 activation by LPS and has been proposed to target TLR4 adaptors MAL/TIRAP and TRAM, since it interacted with both MAL/TIRAP and TRAM although many TLR4-inhibitory peptides target the agonist rather than the receptor [118-121].

##### 2.3.1.3 Peptidomimetics and Small Molecule Inhibitors of TIR-TIR Interactions

To achieve better stability, internalization, pharmacological properties and receptor selectivity, peptidomimetics are synthesized which mimic peptide inhibitors. The aim of peptidomimetics is to maintain the specific molecular interactions between protein-peptide partners while replacing the peptide bonds. The BB loop of TIR domain presented a starting point for developing peptidomimetics as novel therapeutic agents even before BB loop decoy peptides emerged. A low molecular weight molecule hydrocinnamoyl-L-valyl pyrrolidine (compound 4a) based on the sequence of the MyD88 TIR BB loop interfered with the interactions between mouse MyD88 and IL-1RI resulting in selective inhibition of IL1R signaling but not LPS-induced TLR signaling. Hydrocinnamoyl-L-valyl pyrrolidine was tested also *in vivo* where it significantly attenuated IL-1β induced fever response in adult male C57BL/6 mice [122]. Based on this compound several other BB loop peptidomimetics have been designed and tested. It was shown that in hypothalamic neurons two compounds (EM77 and EM110) demonstrated an anti-inflammatory and neuroprotective potential and were able to selectively inhibit MyD88 dependent but not MyD88 independent signaling [123]. Another example of peptidomimetic modeled on BB loop of MyD88 TIR is compound ST2825. ST2825 specifically inhibits *in vitro* dimerization of MyD88 TIR domains, but not DD domains, leading to the inhibition of IL-1R signaling. Moreover, ST2825 suppressed not only IL-1R signaling, but also MyD88 dependent TLR9 signaling. When orally administered in C57BL/6 mice ST2825 inhibited *in vivo* IL-6 response to IL-1β treatment [124]. More recently, in a murine model of nonreperfused acute myocardial infarction ST2825 *in vivo* protected against left ventricular dilatation and hypertrophy [125]. In systemic lupus erythematosus (SLE) ST2825 abolished production of autoantibodies in peripheral blood mononuclear cells from SLE patients stimulated with TLR9 agonist CpG which enables further human clinical testing [126]. While peptidomimetics act by obscuring interactions between protein partners, some inhibitors can covalently bind to proteins and disrupt interactions. Specific inhibition of protein interactions between TLR4 and adapter molecule MAL/TIRAP or TRAM has been achieved with such small-molecule inhibitor TAK-242 (cyclohexene derivative resatorvid). Among 10 different human TLRs, TAK-242 selectively binds to TLR4 via Cys747 in the TIR domain of TLR4. It is presumed that TAK-242 reacts with TLR4 to form a complex containing a covalently attached cyclohexene ring at position 747 and causes a conformational change or a steric hindrance in the TIR domain of TLR4, affecting the recruitment of adapters to the signaling complex [127, 128]. Besides *in vitro *inhibition of various inflammatory mediators, TAK-242 decreased *in vivo* levels of cytokines and protected mice from LPS-induced lethality in endotoxaemic mouse model [129]. The schematic overview of inhibitory peptides and peptidomimetics reviewed above is shown in Table (**2**).

#### 2.3.2 Inhibitory Proteins Targeting TIR Domain Interactions 

The above described peptides and peptidomimetics exhibit a relatively weak inhibitory potency for the TLR inhibition. In order to avoid the need for high concentrations of these peptides for therapy, protein inhibitors with higher inhibitory potency can be employed for the TLR inhibition. Truncated and/or mutated forms of TLR adapter proteins were reported to inhibit TLR signalization as dominant-negative (dn) mutants. These include adapter TIR domains or point mutants in the adapters. Generally, monomeric adapter TIR domains bind to the target interacting partner of the cognate adapter and prevent binding of the native adapter or form nonfunctional heterodimers with their wild type counterparts. Point mutations in the inhibitory adapters are located in the positions critical for their interactions with other proteins and frequently involve the mutation of the conserved proline in the BB loop significant for TIR domain function. Using *in vitro *assays, the inhibition of several TLRs/IL-1R has been shown with TIR domains of adapters MyD88, MAL/TIRAP and TRIF [37, 130]. Negative regulation of TLR signaling was demonstrated with several mutants of the proline residue from BB loop; TRIF P434H was characterized as a dominant-negative regulator of IFN-β promotor induction triggered by poly(I-C)-TLR3 signaling or LPS-TLR4 signaling while TRAM P116H inhibited LPS-TLR4-mediated NF-κB and IFN-β signaling [18]. The application of protein inhibitors from TLR adapters was tested in *in vitro* cell culture models of several diseases by adenoviral transfection of DNA constructs encoding these inhibitors. TLR2, TLR4 and MyD88 have an important role in the development of atherosclerosis characterized by the accumulation of proinflammatory cytokines, chemokines and matrix metalloproteinases (MMPs) mediated by several different cell types in the endothelium of the artery lumen. On the *ex vivo *cultures of cells isolated from human atherosclerosis plaques the production of several cytokines, inflammatory mediators and metalloproteinases was significantly reduced with dnMyD88 (MyD88 aa 53-296 P56N) [131] where the P56N mutation in the DD of MyD88 is unable to form DD homodimers [132]. Though the dnTRAM construct (TRAM C117H), incapable of homodimerization and heterodimerization with TRIF [18] did not have such a broad impact as dnMyD88 it also reduced NF-κB activity and MMP production in atheroma cell cultures [131]. TLRs also promote the inflammatory and destructive processes in rheumatoid arthritis with adapters MyD88 and MAL/TIRAP having a significant role. In synovial membrane cultures from RA tissue, the expression of dn forms of MyD88 (MyD88 aa 53-296 P56N) and MAL/TIRAP (MAL/TIRAP P125H) significantly down-regulated the production of TNF-α, IL-6, IL-8 and VEGF cytokines and metalloproteinases [133]. Using MAPPIT analysis, the conserved proline 125 mutant of MAL/TIRAP was reported to prevent MAL/TIRAP homodimerization, heterodimerization with MyD88 and interaction of MyD88 with TLR4 [134] suggesting its role for the abrogated TLR signaling. MyD88 may also provide a therapeutic target in type I diabetes mellitus given the important role of IL-1β/MyD88-mediated signaling in this disease. In pancreatic islets β cells, the IL-1β induced production of nitric oxide correlates with β cell apoptosis and/or abrogation of insulin secretion [135]. On the conditionally immortalized βTc-Tet cells the lentivirus mediated expression of dominant negative versions of MyD88 (MyD88 TIR or MyD88 F56N) suppressed IL-1β/IFN-γ induced NO production, increased the resistance of cells to apoptosis and retained their insulin secretory response to glucose [136]. The cell type dependent requirement for different adapters in nonmyeloid versus myeloid cells reveals important implications in terms of therapies targeted at blocking LPS-mediated cell activation with its major role in pathology of sepsis. The LPS-triggered signalization in nonmyeloid synovial fibroblasts (SFs) and umbilical vein endothelial cells (HUVECs) was successfully abrogated with dnMyD88, dnMAL/TIRAP and dnTRAM while the inhibitory effect was not evident in myeloid cells [58, 137]. *In vivo*, the application of dominant negative proteins for therapeutic inhibition has been demonstrated for ischemia/reperfusion (I/R) injury, a medical condition which correlates with NF-κB activation. Blocking the MyD88 mediated pathway by adenoviral introduction of dnMyD88 (MyD88 DD) into myocardium of rats protected from I/R injury by blunting NF-κB and activation of the protective Akt signaling pathway [138].

Revealing the details of the structure and molecular function of TIR-domain containing proteins new challenges are offered for the improvement of the existing TLR protein inhibitors. Structural data uncovered the receptor TIR dimer [46, 56, 139] as a functional signaling scaffold for binding of adapters while it was also shown that MyD88 oligomerizes via its DD and TIR domains [132, 134]. Thus, based on the predicted TIR domain interactions in the signaling complexes the dimerized TIR domains should have higher affinities for each other which would lead to their higher inhibitory potency. Covalently tethered TIR domain dimers confirmed this assumption as they exhibited improved inhibition of TLR signaling compared to monomeric TIR domain inhibitors [36]. The concept of TIR domain dimerization for the construction of improved inhibitors was further exploited by linking TIR domains via coiled-coil segments. Coiled-coil mediated TIR domain dimerization is a successful strategy used by bacterial TIR domain containing proteins [91-93] utilized for host TLR signaling subversion. The fusion protein GCN-mTIR where the MyD88 TIR domain was linked to the artificial strong coiled-coil segment GCN4-p1 conferred superior inhibition, several orders of magnitude more efficient than monomeric TIR domain inhibitors or inhibitory peptides [36]. GCN-mTIR has an expanded spectrum for a broad range of TLRs/IL-1R and is therefore of particular therapeutic interest as pathogens often engage several TLRs. From the point of potential therapeutic application one should also consider that the selection of a specific TIR domain of an adapter does not always narrowly define the specificity for TLR inhibition [130, 137] which was also observed with inhibition of TLR3 with dimeric MyD88 TIR domain inhibitors [36] indicating a cross-talk between MyD88 and TRIF dependent pathways. As a comprehensive overview the protein inhibitors originating from TLR adapters with their mechanism of inhibition, TLR specificity and application is given in Table (**3**). 

## CONCLUSION

Recently, excessive activation of innate immune signaling has been associated with several inflammatory diseases. Inhibitors targeting molecules of innate immunity therefore represent promising therapeutic agents. Recently, new findings revealed important insight into the TLR signaling mechanisms [34] that enable design of more efficient inhibitors. The ideal inhibitor should have high specificity against the selected targets, low dosage for the effective inhibition, high bioavailability and should be easily delivered in organism. It should also be obtained in large quantities and have sufficient purity to avoid side effects. Protein inhibitors enable strong and specific suppression of signaling due to multiple and specific interactions with their target and in this respect represent a more suitable type of inhibitors than peptide and peptidomimetic inhibitors that sometimes lack the desired specificity. On the other hand, peptidomimetic inhibitors are low molecular weight compounds that are easily translocated into cells while protein size represents a challenge for the delivery. DNA constructs encoding TLR protein inhibitors can be delivered for therapy *in vivo* or *ex vivo* via different routes including lipofection, microinjection, electroporation, microbombardment or viral delivery [138]. However, the delivery of TLR inhibitor as a purified protein instead of the coding DNA represents an approach to overcome the disadvantages of the gene therapy, but introduces a problem of their cost of production. Besides electroporation, microinjection and liposome delivery of proteins into target cells, the intracellular delivery of the protein inhibitor fused to the protein transduction domain represents a promising strategy for therapeutic applications. The employment of different short cationic peptides such as HIV-1 TAT, penetratin, synthetic poly-arginine and PTD-5 segments has been shown for the intracellular delivery of heterologous proteins as well for peptide inhibitors into cells [140]. Therefore, the future development of TLR signaling inhibitors relies on new discoveries of TLR signaling mechanism as well as on new ways of delivering inhibitors to targets.

## Figures and Tables

**Fig. (1):**
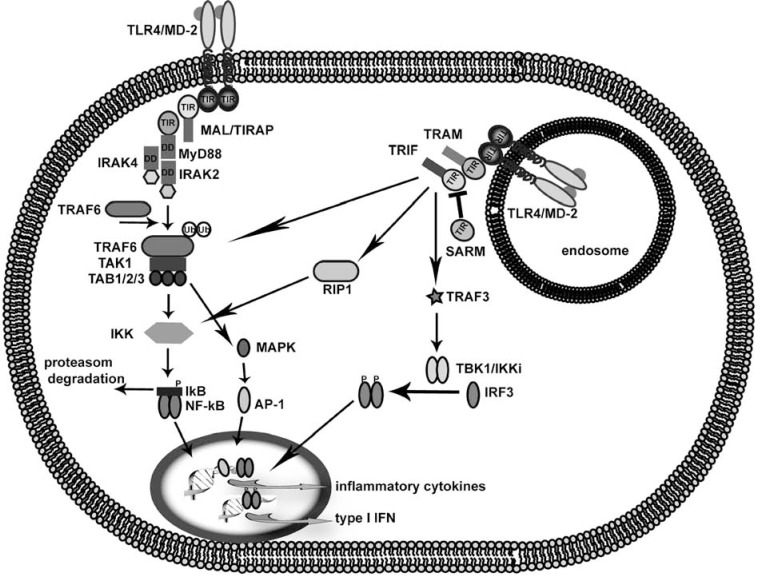
TLR signaling. Schematic representation of MyD88 dependent and TRIF dependent TLR4 signaling pathway culminating in the production of inflammatory cytokines and type I interferons.

**Fig. (2):**
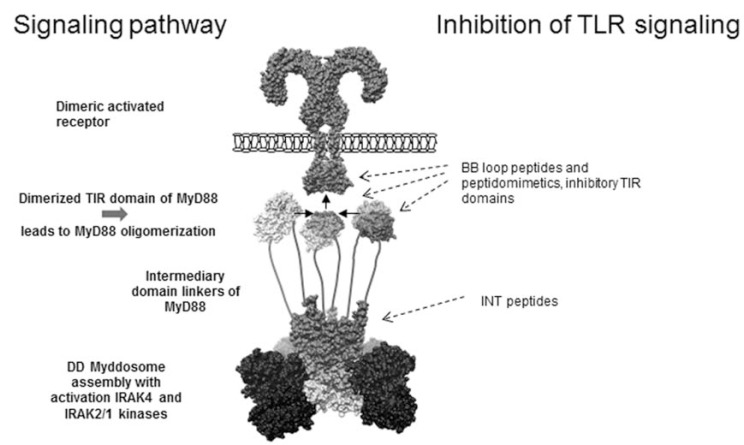
**Molecular model of TLR activation through the dimeric TIR domain platform.** Binding of PAMPs to the ectodomain of the respective TLRs triggers their dimerization. Dimeric TIR of TLR induces binding of a dimer of MyD88 through TIR-TIR domain interaction. Dimeric TIR domain of MyD88 dimer further induces the association of additional MyD88 molecules leading to the assembly of Myddosome with activation of IRAK kinases. The dashed arrows represent the sites for the inhibitory action of peptides, peptidomimetics and inhibitory proteins designated on the right.

**Fig. (3):**
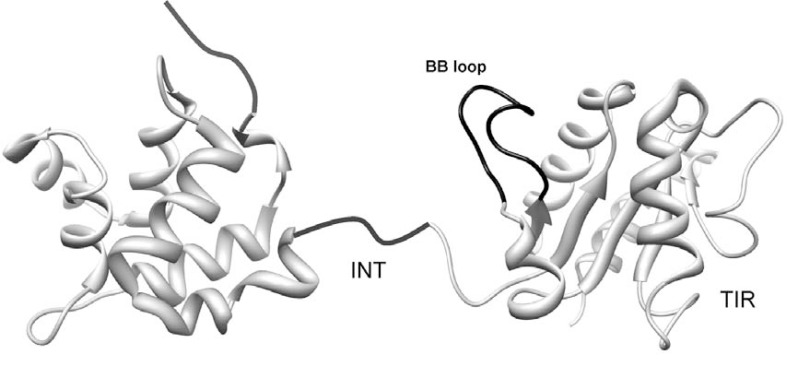
Structure of adapter MyD88. DD (left) and TIR (right) domain are represented as ribbon diagrams based on the tertiary structures of each isolated domain. The designated INT, TIR domain and BB loop represent important regions for inhibitory peptide and protein therapeutics origin.

**Table 1.** Schematic Presentation of Decoy Peptide Composition

Internalization Moiety	Inhibitory Peptide
Cell penetrating peptide: penetratin polyarginine	BB loop of adaptor proteins^1^ inhibitory peptides derived from TIR domain of TLR4, MAL/TIRAP^2^ VIPERIN^3^
Myristoylation	INT domain of MyD88 (INT peptide)^4^

1 [107-109, 114]

2 [110, 111]

3 [116]

4 [34]

**Table 2.** Overview of Inhibitory Peptides and Peptidomimetics

	Source of Inhibitory Sequence/Surface	Effect on Signaling
**INHIBITORY PEPTIDES**		
BB loop peptides	TLR adaptors	inhibition of TLR and IL-1R signaling
peptides from TIR TLR4	TLR4	inhibition of TLR4 signaling
peptides from TIR MAL/TIRAP	MAL/TIRAP	inhibition of TLR4 or TLR2 signaling
VIPERIN	A46	inhibition of TLR4 signaling (VIPERIN targets MAL/TIRAP and TRAM)
INT peptide	MyD88	inhibition of MyD88 dependent signaling (INT peptide targets IRAK4)
**INHIBITORY PEPTIDOMIMETICS**		
hydrocinnamoyl-L-valyl pyrrolidine	MyD88	inhibition of IL-1R signaling
EM77, EM110	MyD88	inhibition of MyD88 dependent signaling
ST2825	MyD88	inhibition of MyD88 dependent signaling

**Table 3.** Overview of TLR/IL-1R Inhibitory Proteins Targeting Intracellular Signaling Pathways

Source Protein	dn Mutant	Proposed Mechanism of Inhibition	Experimentally Established Inhibition	Application
MyD88	TIR	interferes into TIR:TIR interactions	TLR4^1^^,^^2^, TLR5^2^, IL-1R 2^,^^3^	attenuates IL-1β/IFN-γ mediated NO production and apoptosis, retains insulin secretion on pancreatic islets β cells^4^
	DD	interferes into DD:DD interactions		protects from I/R injury by blunting NF-κB and activation of the protective Akt signaling pathway^5^
	MyD88 aa 53-296 P56N	prevents dimerization of MyD88 through DD^6^	TLR4 (nonmieloid cells only), IL-1R^7^	down regulates the production of inflammatory cytokines and metalloproteinases on cultures from human atherosclerotic plaques^8^ and synovial membrane cultures from RA tissue^9^
	MyD88 P56N	prevents dimerization of MyD88 through DD^6^	IL-1R^6^	attenuates IL-1β/IFN-γ mediated NO production and apoptosis, retains insulin secretion on pancreatic islets β cells^4^
	dTIR	interacts with TLR^2^	TLR3, TLR4, TLR5, TLR9, IL-1R^2^	
	GCN-mTIR	interacts with TLR^2^	TLR3, TLR4, TLR5, TLR9, IL-1R^2^	
Mal/TIRAP	TIR	interferes into TIR:TIR interactions	TLR4^10^	down regulate the production of inflammatory cytokines and metalloproteinases on synovial membrane cultures from RA tissue^9^
	Mal P125H	prevents Mal homodimerisation, heterodimerisation with MyD88 and interaction of MyD88 with TLR4^11^	TLR4 (nonmieloid cells only)^7^	
TRIF	TIR	interferes into TIR:TIR interactions	TLR2, TLR3, TLR4, TLR7^12^	
	TRIF P434H	prevents TRIF homodimerisation^13^	TLR3, TLR4^14^	
TRAM	TRAM P116H	prevents TRAM homodimerisation and heterodimerisation with TRIF^14^	TLR4^14^	
	TRAM C117H	prevents TRAM homodimerisation and heterodimerisation with TRIF^14^	TLR2, TLR4 (nonmieloid cells only)^15^	reduces NF-κB and metalloproteinases production in atheroma cell cultures^8^

1 [23, 37],

2 [36],

3 [37, 54],

4 [136],

5 [138],

6 [132],

7 [58],

8 [131],

9 [133],

10 [37],

11 [134],

12 [130],

13 [141],

14 [18],

15 [137].

## References

[1] Beutler B (2004). Innate immunity: an overview. Mol. Immunol.

[2] Janeway C A, Medzhitov R (2002). Innate immune recognition. Annu. Rev. Immunol.

[3] Janeway C A (1989). Approaching the asymptote? Evolution and
revolution in immunology. Cold Spring Harb Symp. Quant. Biol.

[4] Akira S, Takeda K (2004). Toll-like receptor signalling. Nat. Rev. Immunol.

[5] Rock F L, Hardiman G, Timans J C, Kastelein R A, Bazan J F (1998). A family of human receptors structurally related to Drosophila Toll. Proc. Natl. Acad. Sci. USA.

[6] Medzhitov R, Preston-Hurlburt P, Janeway C A (1997). A human homologue of the Drosophila Toll protein signals activation of adaptive immunity. Nature.

[7] Akira S, Uematsu S, Takeuchi O (2006). Pathogen recognition and innate immunity. Cell.

[8] Ishii K J, Coban C, Akira S (2005). Manifold mechanisms of Toll-like receptor-ligand recognition. J. Clin. Immunol.

[9] Gay N J, Gangloff M (2007). Structure and function of Toll receptors and their ligands. Annu. Rev. Biochem.

[10] Jerala R (2007). Structural biology of the LPS recognition. Int. J. Med. Microbiol.

[11] Kim H M, Park B S, Kim J I, Kim S E, Lee J, Oh S C, Enkhbayar P, Matsushima N, Lee H, Yoo O J, Lee J O (2007). Crystal structure of the TLR4-MD-2 complex with bound endotoxin antagonist Eritoran. Cell.

[12] Park B S, Song D H, Kim H M, Choi B S, Lee H, Lee J O (2009). The structural basis of lipopolysaccharide recognition by the TLR4-MD-2 complex. Nature.

[13] Gay N J, Keith F J (1991). Drosophila Toll and IL-1 receptor. Nature.

[14] Schneider D S, Hudson K L, Lin T Y, Anderson K V (1991). Dominant and recessive mutations define functional domains of Toll, a transmembrane protein required for dorsal-ventral polarity in the Drosophila embryo. Genes Dev.

[15] Slack J L, Schooley K, Bonnert T P, Mitcham J L, Qwarnstrom E E, Sims J E, Dower S K (2000). Identification of two major sites in the type I interleukin-1 receptor cytoplasmic region responsible for coupling to pro-inflammatory signaling pathways. J. Biol. Chem.

[16] O'Neill L A, Bowie A G (2007). The family of five: TIR-domain-containing adaptors in Toll-like receptor signalling. Nat. Rev. Immunol.

[17] Yamamoto M, Sato S, Hemmi H, Uematsu S, Hoshino K, Kaisho T, Takeuchi O, Takeda K, Akira S (2003). TRAM is specifically involved in the Toll-like receptor 4-mediated MyD88-independent signaling pathway. Nat. Immunol.

[18] Oshiumi H, Sasai M, Shida K, Fujita T, Matsumoto M, Seya T (2003). TIR-containing adapter molecule (TICAM)-2, a bridging adapter recruiting to toll-like receptor 4 TICAM-1 that induces interferon-beta. J. Biol. Chem.

[19] Yamamoto M, Sato S, Hemmi H, Sanjo H, Uematsu S, Kaisho T, Hoshino K, Takeuchi O, Kobayashi M, Fujita T, Takeda K, Akira S (2002). Essential role for TIRAP in activation of the signalling cascade shared by TLR2 and TLR4. Nature.

[20] Horng T, Barton G M, Medzhitov R (2001). TIRAP: an adapter molecule in the Toll signaling pathway. Nat. Immunol.

[21] Carty M, Goodbody R, Schroder M, Stack J, Moynagh P N, Bowie A G (2006). The human adaptor SARM negatively regulates adaptor protein TRIF-dependent Toll-like receptor signaling. Nat. Immunol.

[22] Kawai T, Adachi O, Ogawa T, Takeda K, Akira S (1999). Unresponsiveness of MyD88-deficient mice to endotoxin. Immunity.

[23] Medzhitov R, Preston-Hurlburt P, Kopp E, Stadlen A, Chen C, Ghosh S, Janeway C A (1998). MyD88 is an adaptor protein in the hToll/IL-1 receptor family signaling pathways. Mol. Cell.

[24] Takeuchi O, Takeda K, Hoshino K, Adachi O, Ogawa T, Akira S (2000). Cellular responses to bacterial cell wall components are mediated through MyD88-dependent signaling cascades. Int. Immunol.

[25] Yamamoto M, Sato S, Hemmi H, Hoshino K, Kaisho T, Sanjo H, Takeuchi O, Sugiyama M, Okabe M, Takeda K, Akira S (2003). Role of adaptor TRIF in the MyD88-independent toll-like receptor signaling pathway. Science.

[26] Honda K, Yanai H, Mizutani T, Negishi H, Shimada N, Suzuki N, Ohba Y, Takaoka A, Yeh W C, Taniguchi T (2004). Role of a transductional-transcriptional processor complex involving MyD88 and IRF-7 in Toll-like receptor signaling. Proc. Natl. Acad. Sci. USA.

[27] Sato S, Sugiyama M, Yamamoto M, Watanabe Y, Kawai T, Takeda K, Akira S (2003). Toll/IL-1 receptor domain-containing adaptor inducing IFN-beta (TRIF) associates with TNF receptor-associated factor 6 and TANK-binding kinase 1, and activates two distinct transcription factors, NF-kappa B and IFN-regulatory factor-3, in the Toll-like receptor signaling. J. Immunol.

[28] Oshiumi H, Matsumoto M, Funami K, Akazawa T, Seya T (2003). TICAM-1, an adaptor molecule that participates in Toll-like receptor 3-mediated interferon-beta induction. Nat. Immunol.

[29] Fitzgerald K A, McWhirter S M, Faia K L, Rowe D C, Latz E, Golenbock D T, Coyle A J, Liao S M, Maniatis T (2003). IKKepsilon and TBK1 are essential components of the IRF3 signaling pathway. Nat. Immunol.

[30] Ohnishi H, Tochio H, Kato Z, Orii K E, Li A, Kimura T, Hiroaki H, Kondo N, Shirakawa M (2009). Structural basis for the multiple interactions of the MyD88 TIR domain in TLR4 signaling. Proc. Natl. Acad. Sci. USA.

[31] Kollewe C, Mackensen A C, Neumann D, Knop J, Cao P, Li S, Wesche H, Martin M U (2004). Sequential autophosphorylation steps in the interleukin-1 receptor-associated kinase-1 regulate its availability as an adapter in interleukin-1 signaling. J. Biol. Chem.

[32] Burns K, Janssens S, Brissoni B, Olivos N, Beyaert R, Tschopp J (2003). Inhibition of interleukin 1 receptor/Toll-like receptor signaling through the alternatively spliced, short form of MyD88 is due to its failure to recruit IRAK-4. J. Exp. Med.

[33] Janssens S, Burns K, Tschopp J, Beyaert R (2002). Regulation of interleukin-1- and lipopolysaccharide-induced NF-kappaB activation by alternative splicing of MyD88. Curr. Biol.

[34] Avbelj M, Horvat S, Jerala R (2011). The role of intermediary domain of MyD88 in cell activation and therapeutic inhibition of TLRs. J. Immunol.

[35] Lin S C, Lo Y C, Wu H (2010). Helical assembly in the MyD88-IRAK4-IRAK2 complex in TLR/IL-1R signalling. Nature.

[36] Fekonja O, Bencina M, Jerala R (2012). Toll/interleukin-1 receptor domain dimers as the platform for activation and enhanced inhibition of Toll-like receptor signaling. J. Biol. Chem.

[37] Fitzgerald K A, Palsson-McDermott E M, Bowie A G, Jefferies C A, Mansell A S, Brady G, Brint E, Dunne A, Gray P, Harte M T, McMurray D, Smith D E, Sims J E, Bird T A, O'Neill L A (2001). Mal (MyD88-adapter-like) is required for Toll-like receptor-4 signal transduction. Nature.

[38] Dunne A, Ejdeback M, Ludidi P L, O'Neill L A, Gay N J (2003). Structural complementarity of Toll/interleukin-1 receptor domains in Toll-like receptors and the adaptors Mal and MyD88. J. Biol. Chem.

[39] Valkov E, Stamp A, Dimaio F, Baker D, Verstak B, Roversi P, Kellie S, Sweet M J, Mansell A, Gay N J, Martin J L, Kobe B (2011). Crystal structure of Toll-like receptor adaptor MAL/TIRAP reveals the molecular basis for signal transduction and disease protection. Proc. Natl. Acad. Sci. USA.

[40] Lin Z, Lu J, Zhou W, Shen Y (2012). Structural insights into TIR domain specificity of the bridging adaptor Mal in TLR4 signaling. PLoS One.

[41] Jiang Z, Mak T W, Sen G, Li X (2004). Toll-like receptor 3-mediated activation of NF-kappaB and IRF3 diverges at Toll-IL-1 receptor domain-containing adapter inducing IFN-beta. Proc. Natl. Acad. Sci. USA.

[42] Kagan J C, Su T, Horng T, Chow A, Akira S, Medzhitov R (2008). TRAM couples endocytosis of Toll-like receptor 4 to the induction of interferon-beta. Nat. Immunol.

[43] Rowe D C, McGettrick A F, Latz E, Monks B G, Gay N J, Yamamoto M, Akira S, O'Neill L A, Fitzgerald K A, Golenbock D T (2006). The myristoylation of TRIF-related adaptor molecule is essential for Toll-like receptor 4 signal transduction. Proc. Natl. Acad. Sci. USA.

[44] Zhang Q, Zmasek C M, Cai X, Godzik A (2011). TIR domain-containing adaptor SARM is a late addition to the ongoing microbe-host dialog. Dev. Comp. Immunol.

[45] Xu Y, Tao X, Shen B, Horng T, Medzhitov R, Manley J L, Tong L (2000). Structural basis for signal transduction by the Toll/interleukin-1 receptor domains. Nature.

[46] Nyman T, Stenmark P, Flodin S, Johansson I, Hammarstrom M, Nordlund P (2008). The crystal structure of the human toll-like receptor 10 cytoplasmic domain reveals a putative signaling dimer. J. Biol. Chem.

[47] Khan J A, Brint E K, O'Neill L A, Tong L (2004). Crystal structure of the Toll/interleukin-1 receptor domain of human IL-1RAPL. J. Biol. Chem.

[48] Panter G, Jerala R (2011). The ectodomain of the Toll-like receptor 4 prevents constitutive receptor activation. J. Biol. Chem.

[49] Underhill D M, Ozinsky A, Hajjar A M, Stevens A, Wilson C B, Bassetti M, Aderem A (1999). The Toll-like receptor 2 is recruited to macrophage phagosomes and discriminates between pathogens. Nature.

[50] Poltorak A, He X, Smirnova I, Liu M Y, Van Huffel C, Du X, Birdwell D, Alejos E, Silva M, Galanos C, Freudenberg M, Ricciardi-Castagnoli P, Layton B, Beutler B (1998). Defective LPS signaling in C3H/HeJ and C57BL/10ScCr mice: mutations in Tlr4 gene. Science.

[51] Fitzgerald K A, Rowe D C, Barnes B J, Caffrey D R, Visintin A, Latz E, Monks B, Pitha P M, Golenbock D T (2003). LPS-TLR4 signaling to IRF-3/7 and NF-kappaB involves the toll adapters TRAM and TRIF. J. Exp. Med.

[52] Rhee S H, Hwang D (2000). Murine TOLL-like receptor 4 confers lipopolysaccharide responsiveness as determined by activation of NF kappa B and expression of the inducible cyclooxygenase. J. Biol. Chem.

[53] Ronni T, Agarwal V, Haykinson M, Haberland M E, Cheng G, Smale S T (2003). Common interaction surfaces of the toll-like receptor 4 cytoplasmic domain stimulate multiple nuclear targets. Mol. Cell Biol.

[54] Li C, Zienkiewicz J, Hawiger J (2005). Interactive sites in the MyD88 Toll/interleukin (IL) 1 receptor domain responsible for coupling to the IL1beta signaling pathway. J. Biol. Chem.

[55] Jiang Z, Georgel P, Li C, Choe J, Crozat K, Rutschmann S, Du X, Bigby T, Mudd S, Sovath S, Wilson I A, Olson A, Beutler B (2006). Details of Toll-like receptor:adapter interaction revealed by germ-line mutagenesis. Proc. Natl. Acad. Sci. USA.

[56] Nunez Miguel R, Wong J, Westoll J F, Brooks H J, O'Neill L A, Gay N J, Bryant C E, Monie T P (2007). A dimer of the Toll-like receptor 4 cytoplasmic domain provides a specific scaffold for the recruitment of signalling adaptor proteins. PLoS One.

[57] Cohen J (2002). The immunopathogenesis of sepsis. Nature.

[58] Andreakos E, Foxwell B, Feldmann M (2004). Is targeting Toll-like receptors and their signaling pathway a useful therapeutic approach to modulating cytokine-driven inflammation?. Immunol. Rev.

[59] Ulevitch R J (2004). Therapeutics targeting the innate immune system. Nat. Rev. Immunol.

[60] Joosten L A, Koenders M I, Smeets R L, Heuvelmans-Jacobs M, Helsen M M, Takeda K, Akira S, Lubberts E, van de Loo F A, van den Berg W B (2003). Toll-like receptor 2 pathway drives streptococcal cell wall-induced joint inflammation: critical role of myeloid differentiation factor 88. J. Immunol.

[61] Shishido T, Nozaki N, Yamaguchi S, Shibata Y, Nitobe J, Miyamoto T, Takahashi H, Arimoto T, Maeda K, Yamakawa M, Takeuchi O, Akira S, Takeishi Y, Kubota I (2003). Toll-like receptor-2 modulates ventricular remodeling after myocardial infarction. Circulation.

[62] Oyama J, Blais C, Liu X, Pu M, Kobzik L, Kelly R A, Bourcier T (2004). Reduced myocardial ischemia-reperfusion injury in toll-like receptor 4-deficient mice. Circulation.

[63] Michelsen K S, Wong M H, Shah P K, Zhang W, Yano J, Doherty T M, Akira S, Rajavashisth T B, Arditi M (2004). Lack of Toll-like receptor 4 or myeloid differentiation factor 88 reduces atherosclerosis and alters plaque phenotype in mice deficient in apolipoprotein E. Proc. Natl. Acad. Sci. USA.

[64] Bjorkbacka H, Kunjathoor V V, Moore K J, Koehn S, Ordija C M, Lee M A, Means T, Halmen K, Luster A D, Golenbock D T, Freeman M W (2004). Reduced atherosclerosis in MyD88-null mice links elevated serum cholesterol levels to activation of innate immunity signaling pathways. Nat. Med.

[65] Kiechl S, Lorenz E, Reindl M, Wiedermann C J, Oberhollenzer F, Bonora E, Willeit J, Schwartz D A (2002). Toll-like receptor 4 polymorphisms and atherogenesis. N. Engl. J. Med.

[66] Marshak-Rothstein A (2006). Toll-like receptors in systemic autoimmune disease. Nat. Rev. Immunol.

[67] Leadbetter E A, Rifkin I R, Hohlbaum A M, Beaudette B C, Shlomchik M J, Marshak-Rothstein A (2002). Chromatin-IgG complexes activate B cells by dual engagement of IgM and Toll-like receptors. Nature.

[68] Couillin I, Vasseur V, Charron S, Gasse P, Tavernier M, Guillet J, Lagente V, Fick L, Jacobs M, Coelho F R, Moser R, Ryffel B (2009). IL-1R1/MyD88 signaling is critical for elastase-induced lung inflammation and emphysema. J. Immunol.

[69] Chen C J, Shi Y, Hearn A, Fitzgerald K, Golenbock D, Reed G, Akira S, Rock K L (2006). MyD88-dependent IL-1 receptor signaling is essential for gouty inflammation stimulated by monosodium urate crystals. J. Clin. Invest.

[70] Martinon F, Petrilli V, Mayor A, Tardivel A, Tschopp J (2006). Gout-associated uric acid crystals activate the NALP3 inflammasome. Nature.

[71] Wen L, Ley R E, Volchkov P Y, Stranges P B, Avanesyan L, Stonebraker A C, Hu C, Wong F S, Szot G L, Bluestone J A, Gordon J I, Chervonsky A V (2008). Innate immunity and intestinal microbiota in the development of Type 1 diabetes. Nature.

[72] Kleinridders A, Schenten D, Konner A C, Belgardt B F, Mauer J, Okamura T, Wunderlich F T, Medzhitov R, Bruning J C (2009). MyD88 signaling in the CNS is required for development of fatty acid-induced leptin resistance and diet-induced obesity. Cell Metab.

[73] Alfonso-Loeches S, Pascual-Lucas M, Blanco A M, Sanchez-Vera I, Guerri C (2010). Pivotal role of TLR4 receptors in alcohol-induced neuroinflammation and brain damage. J. Neurosci.

[74] Racke M K, Drew P D (2009). Toll-like receptors in multiple sclerosis. Curr. Top. Microbiol. Immunol.

[75] Kitazawa M, Cheng D, Tsukamoto M R, Koike M A, Wes P D, Vasilevko V, Cribbs D H, LaFerla F M (2011). Blocking IL-1 signaling rescues cognition, attenuates tau pathology, and restores neuronal beta-catenin pathway function in an Alzheimer's disease model. J. Immunol.

[76] Huang B, Zhao J, Li H, He K L, Chen Y, Chen S H, Mayer L, Unkeless J C, Xiong H (2005). Toll-like receptors on tumor cells facilitate evasion of immune surveillance. Cancer Res.

[77] Kelly M G, Alvero A B, Chen R, Silasi D A, Abrahams V M, Chan S, Visintin I, Rutherford T, Mor G (2006). TLR-4 signaling promotes tumor growth and paclitaxel chemoresistance in ovarian cancer. Cancer Res.

[78] Rakoff-Nahoum S, Medzhitov R (2007). Regulation of spontaneous intestinal tumorigenesis through the adaptor protein MyD88. Science.

[79] Kanzler H, Barrat F J, Hessel E M, Coffman R L (2007). Therapeutic targeting of innate immunity with Toll-like receptor agonists and antagonists. Nat. Med.

[80] Rossignol D P, Lynn M (2005). TLR4 antagonists for endotoxemia and beyond. Curr. Opin. Investig. Drugs.

[81] Spiller S, Elson G, Ferstl R, Dreher S, Mueller T, Freudenberg M, Daubeuf B, Wagner H, Kirschning C J (2008). TLR4-induced IFN-gamma production increases TLR2 sensitivity and drives Gram-negative sepsis in mice. J. Exp. Med.

[82] Kalia S, Dutz J P (2007). New concepts in antimalarial use and mode of action in dermatology. Dermatol. Ther.

[83] Kim W U, Sreih A, Bucala R (2009). Toll-like receptors in systemic lupus erythematosus; prospects for therapeutic intervention. Autoimmun. Rev.

[84] Barrat F J, Meeker T, Chan J H, Guiducci C, Coffman R L (2007). Treatment of lupus-prone mice with a dual inhibitor of TLR7 and TLR9 leads to reduction of autoantibody production and amelioration of disease symptoms. Eur. J. Immunol.

[85] Karin M, Yamamoto Y, Wang Q M (2004). The IKK NF-kappa B system: a treasure trove for drug development. Nat. Rev. Drug Discov.

[86] Karin M (2005). Inflammation-activated protein kinases as targets for drug development. Proc. Am. Thorac. Soc.

[87] Wang Z, Wesche H, Stevens T, Walker N, Yeh W C (2009). IRAK-4 inhibitors for inflammation. Curr. Top. Med. Chem.

[88] Brint E K, Xu D, Liu H, Dunne A, McKenzie A N, O'Neill L A, Liew F Y (2004). ST2 is an inhibitor of interleukin 1 receptor and Toll-like receptor 4 signaling and maintains endotoxin tolerance. Nat. Immunol.

[89] Qin J Z, Qian Y C, Yao J H, Grace C, Li X X (2005). SIGIRR inhibits interleukin-1 receptor- and toll-like receptor 4-mediated signaling through different mechanisms. J. Biol. Chem.

[90] Mansell A, Smith R, Doyle S L, Gray P, Fenner J E, Crack P J, Nicholson S E, Hilton D J, O'Neill L A, Hertzog P J (2006). Suppressor of cytokine signaling 1 negatively regulates Toll-like receptor signaling by mediating Mal degradation. Nat. Immunol.

[91] Bowie A, Kiss-Toth E, Symons J A, Smith G L, Dower S K, O'Neill L A (2000). A46R and A52R from vaccinia virus are antagonists of host IL-1 and toll-like receptor signaling. Proc. Natl. Acad. Sci. USA.

[92] Cirl C, Wieser A, Yadav M, Duerr S, Schubert S, Fischer H, Stappert D, Wantia N, Rodriguez N, Wagner H, Svanborg C, Miethke T (2008). Subversion of Toll-like receptor signaling by a unique family of bacterial Toll/interleukin-1 receptor domain-containing proteins. Nat. Med.

[93] Newman R M, Salunkhe P, Godzik A, Reed J C (2006). Identification and characterization of a novel bacterial virulence factor that shares homology with mammalian Toll/interleukin-1 receptor family proteins. Infect. Immun.

[94] Gradisar H, Keber M M, Pristovsek P, Jerala R (2007). MD-2 as the target of curcumin in the inhibition of response to LPS. J. Leukoc. Biol.

[95] Mancek-Keber M, Gradisar H, Inigo Pestana M, Martinez de Tejada G, Jerala R (2009). Free thiol group of MD-2 as the target for inhibition of the lipopolysaccharide-induced cell activation. J. Biol. Chem.

[96] Mancek-Keber M, Jerala R (2006). Structural similarity between the hydrophobic fluorescent probe and lipid A as a ligand of MD-2. FASEB J.

[97] Resman N, Gradisar H, Vasl J, Keber M M, Pristovsek P, Jerala R (2008). Taxanes inhibit human TLR4 signaling by binding to MD-2. FEBS Lett.

[98] Kerkis A, Hayashi M A, Yamane T, Kerkis I (2006). Properties of cell penetrating peptides (CPPs). IUBMB Life.

[99] Futaki S, Suzuki T, Ohashi W, Yagami T, Tanaka S, Ueda K, Sugiura Y (2001). Arginine-rich peptides. An abundant source of
membrane-permeable peptides having potential as carriers for
intracellular protein delivery. J. Biol. Chem.

[100] Kalderon D, Roberts B L, Richardson W D, Smith A E (1984). A short amino acid sequence able to specify nuclear location. Cell.

[101] Schutze-Redelmeier M P, Gournier H, Garcia-Pons F, Moussa M, Joliot A H, Volovitch M, Prochiantz A, Lemonnier F A (1996). Introduction of exogenous antigens into the MHC class I processing and presentation pathway by Drosophila antennapedia homeodomain primes cytotoxic T cells in vivo. J. Immunol.

[102] Schwarze S R, Ho A, Vocero-Akbani A, Dowdy S F (1999). In vivo protein transduction: delivery of a biologically active protein into the mouse. Science.

[103] Nir S, Nieva J L (2000). Interactions of peptides with liposomes: pore formation and fusion. Prog. Lipid Res.

[104] Mishra A, Lai G H, Schmidt N W, Sun V Z, Rodriguez A R, Tong R, Tang L, Cheng J, Deming T J, Kamei D T, Wong G C (2011). Translocation of HIV TAT peptide and analogues induced by multiplexed membrane and cytoskeletal interactions. Proc. Natl. Acad. Sci. USA.

[105] Nelson A R, Borland L, Allbritton N L, Sims C E (2007). Myristoyl-based transport of peptides into living cells. Biochemistry.

[106] Letoha T, Kusz E, Papai G, Szabolcs A, Kaszaki J, Varga I, Takacs T, Penke B, Duda E (2006). In vitro and in vivo nuclear factor-kappaB inhibitory effects of the cell-penetrating penetratin peptide. Mol. Pharmacol.

[107] Toshchakov V U, Basu S, Fenton M J, Vogel S N (2005). Differential involvement of BB loops of toll-IL-1 resistance (TIR) domain-containing adapter proteins in TLR4- versus TLR2-mediated signal transduction. J. Immunol.

[108] Toshchakov V Y, Fenton M J, Vogel S N (2007). Cutting Edge: Differential inhibition of TLR signaling pathways by cell-permeable peptides representing BB loops of TLRs. J. Immunol.

[109] Toshchakov V Y, Vogel S N (2007). Cell-penetrating TIR BB loop decoy peptides a novel class of TLR signaling inhibitors and a tool to study topology of TIR-TIR interactions. Expert. Opin. Biol. Ther.

[110] Toshchakov V Y, Szmacinski H, Couture L A, Lakowicz J R, Vogel S N (2011). Targeting TLR4 signaling by TLR4 Toll/IL-1 receptor domain-derived decoy peptides: identification of the TLR4 Toll/IL-1 receptor domain dimerization interface. J. Immunol.

[111] Couture L A, Piao W, Ru L W, Vogel S N, Toshchakov V Y (2012). Targeting Toll-like receptor (TLR) signaling by Toll/IL-1R domain-containing adapter protein/MyD88-adapter-like- (TIRAP/Mal-) derived decoy peptides. J. Biol. Chem.

[112] Jeyaseelan S, Manzer R, Young S K, Yamamoto M, Akira S, Mason R J, Worthen G S (2005). Toll-IL-1 receptor domain-containing adaptor protein is critical for early lung immune responses against Escherichia coli lipopolysaccharide and viable Escherichia coli. J. Immunol.

[113] Loiarro M, Ruggiero V, Sette C (2010). Targeting TLR/IL-1R signalling in human diseases. Mediators Inflamm.

[114] Loiarro M, Sette C, Gallo G, Ciacci A, Fanto N, Mastroianni D, Carminati P, Ruggiero V (2005). Peptide-mediated interference of TIR domain dimerization in MyD88 inhibits interleukin-1-dependent activation of NF-{kappa}B. J. Biol. Chem.

[115] Egunsola A T, Zawislak C L, Akuffo A A, Chalmers S A, Ewer J C, Vail C M, Lombardo J C, Perez D N, Kurt R A (2012). Growth, metastasis, and expression of CCL2 and CCL5 by murine mammary carcinomas are dependent upon Myd88. Cell Immunol.

[116] Lysakova-Devine T, Keogh B, Harrington B, Nagpal K, Halle A, Golenbock D T, Monie T, Bowie A G (2010). Viral inhibitory peptide of TLR4, a peptide derived from vaccinia protein A46, specifically inhibits TLR4 by directly targeting MyD88 adaptor-like and TRIF-related adaptor molecule. J. Immunol.

[117] Stack J, Haga I R, Schroder M, Bartlett N W, Maloney G, Reading P C, Fitzgerald K A, Smith G L, Bowie A G (2005). Vaccinia virus protein A46R targets multiple Toll-like-interleukin-1 receptor adaptors and contributes to virulence. J. Exp. Med.

[118] Japelj B, Pristovsek P, Majerle A, Jerala R (2005). Structural origin of endotoxin neutralization and antimicrobial activity of a lactoferrin-based peptide. J. Biol. Chem.

[119] Mancek M, Pristovsek P, Jerala R (2002). Identification of LPS-binding peptide fragment of MD-2, a toll-receptor accessory protein. Biochem. Biophys. Res. Commun.

[120] Mancek-Keber M, Bencina M, Japelj B, Panter G, Andra J, Brandenburg K, Triantafilou M, Triantafilou K, Jerala R (2012). MARCKS as a negative regulator of lipopolysaccharide signaling. J. Immunol.

[121] Zorko M, Japelj B, Hafner-Bratkovic I, Jerala R (2009). Expression, purification and structural studies of a short antimicrobial peptide. Biochim. Biophys. Acta.

[122] Bartfai T, Behrens M M, Gaidarova S, Pemberton J, Shivanyuk A, Rebek J (2003). A low molecular weight mimic of the Toll/IL-1 receptor/resistance domain inhibits IL-1 receptor-mediated responses. Proc. Natl. Acad. Sci. USA.

[123] Davis C N, Mann E, Behrens M M, Gaidarova S, Rebek M, Rebek J, Bartfai T (2006). MyD88-dependent and -independent signaling by IL-1 in neurons probed by bifunctional Toll/IL-1 receptor domain/BB-loop mimetics. Proc. Natl. Acad. Sci. USA.

[124] Loiarro M, Capolunghi F, Fanto N, Gallo G, Campo S, Arseni B, Carsetti R, Carminati P, De Santis R, Ruggiero V, Sette C (2007). Pivotal Advance: Inhibition of MyD88 dimerization and recruitment of IRAK1 and IRAK4 by a novel peptidomimetic compound. J. Leukoc. Biol.

[125] Van Tassell B W, Seropian I M, Toldo S, Salloum F N, Smithson L, Varma A, Hoke N N, Gelwix C, Chau V, Abbate A (2010). Pharmacologic inhibition of myeloid differentiation factor 88 (MyD88) prevents left ventricular dilation and hypertrophy after experimental acute myocardial infarction in the mouse. J. Cardiovasc. Pharmacol.

[126] Capolunghi F, Rosado M M, Cascioli S, Girolami E, Bordasco S, Vivarelli M, Ruggiero B, Cortis E, Insalaco A, Fanto N, Gallo G, Nucera E, Loiarro M, Sette C, De Santis R, Carsetti R, Ruggiero V (2010). Pharmacological inhibition of TLR9 activation blocks autoantibody production in human B cells from SLE patients. Rheumatol. (Oxford).

[127] Kawamoto T, Ii M, Kitazaki T, Iizawa Y, Kimura H (2008). TAK-242 selectively suppresses Toll-like receptor 4-signaling mediated by the intracellular domain. Eur. J. Pharmacol.

[128] Matsunaga N, Tsuchimori N, Matsumoto T, Ii M (2011). TAK-242 (resatorvid), a small-molecule inhibitor of Toll-like receptor (TLR) 4 signaling, binds selectively to TLR4 and interferes with interactions between TLR4 and its adaptor molecules. Mol. Pharmacol.

[129] Sha T, Sunamoto M, Kitazaki T, Sato J, Ii M, Iizawa Y (2007). Therapeutic effects of TAK-242, a novel selective Toll-like receptor 4 signal transduction inhibitor, in mouse endotoxin shock model. Eur. J. Pharmacol.

[130] Yamamoto M, Sato S, Mori K, Hoshino K, Takeuchi O, Takeda K, Akira S (2002). Cutting edge: a novel Toll/IL-1 receptor domain-containing adapter that preferentially activates the IFN-beta promoter in the Toll-like receptor signaling. J. Immunol.

[131] Monaco C, Gregan S M, Navin T J, Foxwell B M, Davies A H, Feldmann M (2009). Toll-like receptor-2 mediates inflammation and matrix degradation in human atherosclerosis. Circulation.

[132] Burns K, Martinon F, Esslinger C, Pahl H, Schneider P, Bodmer J L, Di Marco F, French L, Tschopp J (1998). MyD88, an adapter protein involved in interleukin-1 signaling. J. Biol. Chem.

[133] Sacre S M, Andreakos E, Kiriakidis S, Amjadi P, Lundberg A, Giddins G, Feldmann M, Brennan F, Foxwell B M (2007). The Toll-like receptor adaptor proteins MyD88 and Mal/TIRAP contribute to the inflammatory and destructive processes in a human model of rheumatoid arthritis. Am. J. Pathol.

[134] Ulrichts P, Peelman F, Beyaert R, Tavernier J (2007). MAPPIT analysis of TLR adaptor complexes. FEBS Lett.

[135] Mandrup-Poulsen T (1996). The role of interleukin-1 in the pathogenesis of IDDM. Diabetologia.

[136] Dupraz P, Cottet S, Hamburger F, Dolci W, Felley-Bosco E, Thorens B (2000). Dominant negative MyD88 proteins inhibit interleukin-1beta /interferon-gamma -mediated induction of nuclear factor kappa B-dependent nitrite production and apoptosis in beta cells. J. Biol. Chem.

[137] Sacre S M, Lundberg A M, Andreakos E, Taylor C, Feldmann M, Foxwell B M (2007). Selective use of TRAM in lipopolysaccharide (LPS) and lipoteichoic acid (LTA) induced NF-kappaB activation and cytokine production in primary human cells: TRAM is an adaptor for LPS and LTA signaling. J. Immunol.

[138] Hua F, Ha T, Ma J, Gao X, Kelley J, Williams D L, Browder I W, Kao R L, Li C (2005). Blocking the MyD88-dependent pathway protects the myocardium from ischemia/reperfusion injury in rat hearts. Biochem. Biophys. Res. Commun.

[139] Bovijn C, Ulrichts P, De Smet A S, Catteeuw D, Beyaert R, Tavernier J, Peelman F (2012). Identification of interaction sites for dimerization and adapter recruitment in Toll/interleukin-1 receptor (TIR) domain of Toll-like receptor 4. J. Biol. Chem.

[140] Kabouridis P S (2003). Biological applications of protein transduction technology. Trends Biotechnol.

[141] Funami K, Sasai M, Oshiumi H, Seya T, Matsumoto M (2008). Homo-oligomerization is essential for Toll/interleukin-1 receptor domain-containing adaptor molecule-1-mediated NF-kappaB and interferon regulatory factor-3 activation. J. Biol. Chem.

